# Understanding psychoanalytic work online and back to the couch in the wake of the COVID-19 pandemic: an investigation among Italian psychoanalysts

**DOI:** 10.3389/fpsyg.2023.1167582

**Published:** 2023-06-22

**Authors:** Licia Lea Reatto, Andrzej Werbart, Osmano Oasi, Francesca De Salve, Elena Ierardi, Mattia Giordano, Cristina Riva Crugnola

**Affiliations:** ^1^IPA and Italian Psychoanalytic Society, Milan, Italy; ^2^Department of Psychology, Stockholm University, Stockholm, Sweden; ^3^Department of Psychology, Catholic University of the Sacred Heart, Milan, Italy; ^4^Department of Psychology, University of Milano-Bicocca, Milan, Italy

**Keywords:** remote psychoanalysis, COVID-19, attachment style, personality configuration, therapeutic alliance, therapeutic process

## Abstract

**Background:**

Worldwide, psychotherapists’ clinical experience went through rapid developments with transition to teletherapy during the COVID-19 pandemic. Literature on the use of remote psychoanalysis was not conclusive, leaving the issue of the consequences of the necessary setting alternation open. This study aimed to investigate the psychoanalysts’ experiences of shifting to remote work and then returning to in-person setting, considering the effect of the patients’ attachment styles and personality configurations.

**Method:**

Seventy-one analysts of the Italian Psychoanalytic Society were asked to fill out an online survey about patients who found the transition easier and patients who found it more difficult. General questions on therapeutic work, ISTS (Interpretive and Supportive Technique Scale) for interpretive and supportive aspects of technique, WAI-S-TR (Working Alliance Inventory-Short Revised-Therapist) for therapeutic alliance, RQ (Relationship Questionnaire) for attachment style, and PMAI (Prototype Matching of Anaclitic-Introjective Personality Configuration) for personality configurations were administered.

**Results:**

All of the analysts chose to continue the treatment using audio-visual tools. Patients with difficult transitions had a significantly higher frequency of insecure attachment and a higher score on RQ Dismissing scale than patients with easy transitions. No significant differences were found between the two groups in personality configurations, psychotherapeutic alliance, and psychotherapeutic technique. Moreover, a higher level of therapeutic alliance was positively correlated to RQ Secure scale and was negatively correlated to RQ Dismissing scale. Patients with easy transition both to remote work and back to in-person setting had higher scores of therapeutic alliances than those with difficult transition both to remote work and back to in-person setting.

**Conclusion:**

Online psychoanalytic therapy was widely used during the COVID-19 pandemic. Patients with insecure attachment styles had greater difficulties in adapting to setting alternations, thus confirming that insecure attachment is a vulnerability factor not only for psychopathological problems but also for a well-functioning therapeutic collaboration. Patient’s personality configuration did not influence their adaptation to the setting alternation. The supportive and interpretive styles did not undergo significant changes in the transition from in-person setting to remote setting and vice versa, thus suggesting a continuity in the analysts’ “internal setting.”

## 1. Introduction

The severe pandemic, which to different extent spread across the world in 2019, produced a change in human relations. The medical and social measures applied did manage to lead painstakingly to a gradual decrease of the public health danger, although it was not fully resolved. This achievement however did have some severe consequences, which affected everyone’s practical and relational life and, inevitably, individual experience. In general, especially in Italy, one of the first countries that were severely and most harshly hit by the pandemic, there was an almost complete restriction of human contact, limiting it to the strictest necessities for extended periods of time. This and other mandatory behaviors to counteract COVID-19 took on a weight that could give rise to potentially stressful situations, with gradually more severe consequences up to potential traumatic impact (Kumar et al., 2020; Gullo et al., 2021; Preti et al., 2021; Rossi et al., 2022; Cavalera et al., 2023; De Salve et al., 2023). We are still experiencing the repercussions of the pandemic, despite its increasing remission. Only recently did the World Health Organization declare the pandemic over and ease restrictions. In the 2020-23th a series of consequences have affected the psychotherapeutic and psychoanalytic work, called both to collect the sometimes-painful responses of individuals and to deal with new forms of distress that developed in response to the exceptional nature of the pandemic-related situation and the consequences of the restrictions (Gabbard, 2020). The change concerned above all the modes of communication, in particular, the extensive use of synchronous remote communication, which reduces the different ways of contact and enhances the exchange through other perceptual, visual, and acoustic channels – a factor often not too considered. It was necessary to modify first temporarily, then for long periods, the physical co-presence of therapist and patient, previously regulated by a specific setting, as a habitual vehicle of human exchange, essential for the therapeutic process to take place. The majority of analysts and psychotherapists had to resort to a set-up that would allow the continuation of the therapeutic work even remotely, revising the usual methods. The inevitable choice was the use of audio-visual devices, currently quite advanced, already partially in use, without however having systematically tested the consequences in the therapeutic field. Most of the previous experiences in the psychoanalytic area concerned training, with significant results (Fonda, 2011), following a mixed method of alternating between remote and in-person therapy. Other experiences have had unsystematic character, bringing to the fore the question of compatibility with the development of the therapeutic process.

Indeed, it was necessary to resort to remote synchronous activity to accompany patients during a critical time and in order to safeguard the therapeutic continuity in front of the new situation, without the support of previous systematic studies in the psychoanalytic and psychotherapeutic field. In the first period the therapeutic activity was carried out like in an emergency situation, both for the analyst and the patient. It is not by chance that Bolognini (2020) used the image of the use of tents in the event of an earthquake until some understanding of the situation takes place and recovery operations start. We can find an attempt at understanding – initiated by a group of psychoanalysts living and working in Italy – in *Funzione Gamma,* monographic issue that was published a year after Bolognini’s (2020) intervention and focused entirely on this topic (Goisis and Merciai, 2021) by pointing out some risks. In general, there is some agreement that narcissistic and dissociative aspects are most implicated in online use. In the former case we can usefully frame the issue from a sociological point of view, with the now widespread need to have one’s own narcissistic space – on Facebook, Instagram and other social media – in which the identification of the Other is irrelevant (Han, 2015) or more in the background. In the latter we can refer more usefully to the clinical standpoint, where the online can become a “psychic retreat” (Steiner, 2003) of a mind that would otherwise be prey to a sense of inadequacy, anxiety and so on, but can also give rise to actual addiction (Caretti, 2000). Here the importance of the therapeutic relationship comes into play, as a tool that can prevent this kind of risk in the use of online.

In fact, some studies already examined the use of new technologies both in response to increasing social mobility and the extension of psychotherapeutic techniques (Fonda, 2011) and as a reinforcement of therapies in psychiatric settings with patients who find it difficult to tolerate distance (Grenyer, 2013; Jorm et al., 2013). We find two different areas of application: one related to the training of future psychoanalysts in countries without training institutes; the other to specific social or clinical situations. In the former case it is a historical issue, of which the history of psychoanalysis has even some illustrious examples – think of part of Ferenczi’s psychoanalytic treatment with the father of psychoanalysis, Freud, in Vienna – and about which there are, on the whole, rather tolerant stances from International Psychoanalytical Association (IPA) (International Psychoanalytic Association, 2017, 2018). In the latter, the situation is more nuanced, with more and more studies, albeit of an exploratory nature, concerning different psychotherapeutic methods, with inevitable evolutions from the point of view of psychoanalytic technique that are not shared by all clinicians in the field (Marzi, 2021; Nicolò, 2021). Beyond some issues relating to confidence, the question, in the modified setting, concerns the formation and evolution of the therapeutic relationship and the therapeutic alliance, essential for the development of the associative process and therapeutic elaboration (Freud, 1915–1917; Sandler, 1983).

From a more strictly psychoanalytic point of view, the heart of the matter is to answer the following question: does psychoanalysis retain its specificity in relying on online exchange or not? The answers, are not unequivocal. On the one hand, based on established effectiveness, some authors (e.g., Scharff, 2013) have not only come out in favor of online psychoanalysis, but have even gone so far as to argue, despite the inevitable “adjustments” required – that psychoanalysis still retains its specificity even online; “Psychoanalysis is the encounter with an understanding mind in whatever setting that may occur” (Scharff, 2013, p. 8). On the other hand, mainly by considering it unacceptable that the analytical relationship can be “disembodied,” like all virtual relationships, other authors have strongly contested the possibility of teleanalysis (e.g., Argentieri and Mehler, 2003). It seems to be more of a generational conflict than a real conceptual opposition, even if the debate focuses on some aspects that should not be overlooked. For instance, Roesler, a Jungian analyst, warns of the risk of not grasping the non-verbal cues of the relationship, bearers of emotional aspects on which analytic work is often based. From another point of view, Migone (2013) considers it a futile effort to hold together two situations that are different by their nature; online psychoanalysis should not be considered as a mere imitation or simulation of in-person psychoanalysis but should rather be viewed in its specific characteristics. The ever-increasing though exploratory studies have had the merit of testing the appropriateness of adapting the analytical method to remote mode, as well as assessing its possible clinical consequences. The debate did not rule out the possibility of remote use, but indicated the opportunity to explore aspects that could prove decisive: the subjective characteristics of the patient, the development of the alliance and of the therapeutic process; on the technical front, what is lost (non-verbal communications; the transitional aspects studied for example by Werbart et al., 2022a; Aafjes-van Doorn et al., 2022) and what is acquired (repair versus distance; greater knowledge of personal and behavioral aspects in the case of video tools; no discontinuation of therapy) Prompted by all this, the European Psychoanalytical Federation (EPF), which gathers European psychoanalysts and is an integral part of IPA, formed a working group to focus on the issues related to the use of remote treatment, which has given rise to some studies (Marzi, 2021).

The COVID-19 pandemic, under the pressure of the emergency, has caused an intensification of studies on remote therapy, to investigate its practicability and effectiveness. We find two orders of investigation that have taken place in the literature: in the psychoanalytic field an intense study has been developed on the changes that setting modification brought about in the therapeutic relationship, and on the many application fields of the clinical method; the main psychoanalytic concepts and their applications have been revisited in numerous national and international Webinars, which have partly resulted in publications and inspired the theme of the 2023 IPA International Congress. A second order of studies regards the broader field of psychotherapy, with the development of numerous empirical studies aimed at investigating the new situation, with attention to the effects of online therapy and its effectiveness.

Among the relevant observations in the psychoanalytic field, Altman (2020) underlines the physical change that occurs in online therapy as for the physical distance/co-presence of the dyad focusing on the effect on the patient’s attachment system, the analyst’s reflexive function, and, in the change, the role of the body with its instinctual components. Werbart et al. (2022a) have also been investigating on the influence of the attachment system on online transition. With regards to the emergency situation, from different perspectives, Roussillon (2020) and Guignard and Diatkine (2021) focused on the potential regression-provoking effects of the traumatic situation that can enhance dependency, but also on the containing function of the therapeutic relationship regarding the inevitable regressive instances. Erlich’s observation, on the other hand, turned to the distinction between traumatic event and traumatic experience (2021) potentially activated by the pandemic, emphasizing the dual aspect, active and passive, which characterizes the experience, which must be taken into account in the clinical experience. On the one hand, he refers to Freud’s observation, according to which an event can become traumatic in the absence of social containment, and to the anxiety containment function offered by the therapeutic relationship. On the other hand, we also find here a reference to the anaclitic/introjective modality of experience described by Blatt (2008), with whom Erlich also worked (Erlich and Blatt, 1985). In an analogous line of thought, he develops the distinction between ‘internal analytic setting’ and ‘external analytic setting’, supported also by Gampel (2020) and Ehrlich (2021), thus leading to distinguish between setting as a rule and setting as a tool.

We are here introduced to one of the central concepts that have guided our research, the attention paid to personality characteristics, in terms of dependency/autonomy polarity (of relational significance), following Blatt (2008), and their impact on the therapeutic relationship, to assess their influence on the acceptance/lack of acceptance of transitions (transition to teletherapy and return to sessions in person). At the center of our study, and also of all this debate, we place the concept of the therapeutic relationship, essential tool for the development of the therapeutic process, and the related concept of therapeutic alliance, definitely introduced in psychoanalysis by Zetzel (1956; see also Meissner, 1996; Ponsi, 2000), transversely recognized as a variable linked to the outcome (Ackerman and Hilsenroth, 2003; Ardito and Rabellino, 2011). Indeed, we can better speak of a common factor, variously modulated according to the situation.

The debate on online and therapeutic alliance is therefore still very much open: as evidence of this, one can consider the four volumes edited by Scharff (2013, 2015, 2017, 2019) with contributions from psychoanalysts belonging to societies in different parts of the world and about various aspects of teletherapy: from the clinical to the educational realms, from the technical dimension to the transmission of psychoanalytic knowledge. Similarly, some questions on telepsychoanalysis prompted by the pandemic remain unanswered: Are setting modifications compatible with the unfolding of the psychoanalytic process, which considers the relationship essential by using a specific setting to foster the working-through in a relational context (Foresti, 2020; Gabbard, 2020, who emphasized the fragility of the analyst, in a two-person dimension; Gampel, 2020; Puget, 2020)? How did the transition and the subsequent return to in-person setting (due to a decrease in confinement measures) alter the therapeutic relationship and collaboration, and which mechanisms are particularly involved (Kristeva et al., 2020; Levy, 2020)? One further question remains open: For which patients did setting alternation have hindering impacts, and for which it rather facilitated the joint therapeutic work?

The large literature on empirical research that has developed in the wider psychotherapeutic field over the past 3 years, after the onset of the pandemic, has also aimed to answer some of these questions; previous empirical studies are instead quite rare (Cantelmi et al., 2000; Backhaus et al., 2012; Sucala et al., 2012).

Empirical studies during the early stages of the pandemic critical period were delving into the direct experiences of both patients and analysts with regard to remote therapy. Confirming a large recourse to distance therapy, a loss has been described with regard to the framework accompanying treatment (Werbart et al., 2022a), as well as undermined security (Ahlström et al., 2022; Békés and Aafjes-van Doorn, 2022). However, a change has been observed over time; studies after a longer period of time have revealed a greater familiarity with the IT tool and remote work on the part of the therapist, and a decrease in anxiety which also accompanied the patient’s processing work, less burdened by the emergency (*ibidem*).

Our research is placed in this order of reflections and aims to investigate, at a distance of time from the onset and therefore with somewhat consolidated results, the extension of recourse to remote therapy in the Italian psychoanalytic community in the most critical periods of the lockdown, despite the concerns expressed in earlier psychoanalytic literature. The study aims to answer the doubts raised on the development of the therapeutic process in tele-analysis; in particular we wanted to know the vicissitudes of the therapeutic alliance in transition; if the use of online therapy could contain the possible regressive tendencies and the elaboration process has been able to evolve; if the personological characteristics and the attachment system appear to have an effect on the transition, as the literature seems to suggest; whether there have been alterations in interpretive technique; whether or not online therapy ultimately alters the development of the analytic process and how it responds to emergency situations. We wanted to verify these findings on a specific sample and within a longer time frame, to see how they evolved.

### 1.1. Approach to our study

The study aimed at collecting information about both the phase of transition to remote therapy and the phase of return, partially or totally, to in-person setting during the COVID-19 pandemic. The first purpose was to collect information, at a descriptive level, on the analysts’ evaluation of the patients’ experience regarding both the phase of transition and of return, partially or totally, to in-person settings. A second exploratory objective was to test differences between patients with difficult or easy transition to setting changes. Differences concerning variables related to socio-demographic characteristics, duration of treatment, type of problems of the patient, and with respect to attachment style, psychotherapeutic alliance, personality configuration, and psychotherapeutic technique were analyzed. In addition, possible differences between the two groups and types of setting (remote work and return to in-person setting) in terms of psychotherapeutic alliance and psychotherapeutic technique were assessed at an exploratory level.

## 2. Materials and methods

### 2.1. Participants

Approximately one thousand analysts from the thirteen centers of the Italian Psychoanalytic Society were involved with some preparatory meetings and *ad hoc* questionnaires. The data collection was performed entirely online through the Qualtrics platform, after the acquisition of written informed consent, and analysts who voluntarily decided to participate filled in a battery of questionnaires divided into two sections. Eighty-six analysts of the Italian Psychoanalytic Society were involved, 71 completed the questionnaire in the full first section, and 20 completed it in the full second section.

The first section of the survey includes a series of general questions on the development of therapeutic work in different phases of transitions to and from remote work; the second section evaluates specific aspects related to patients in treatment who experienced the transition positively or with difficulty. More specifically, in this section each analyst was asked to answer with two types of patients in mind: those with an easy transition to setting changes, patient A; those with a difficult transition, patient B.

All analysts have an established analytic practice and have completed training at the Italian Psychoanalytic Society.

### 2.2. Procedures

All the study participants gave their informed consent after being properly informed.

The research was authorized by the President of the Italian Psychoanalytic Society within which it was performed and followed the principles of the Declaration of Helsinki.

The research was carried out during the first semester 2022, in a fairly generalized resumption to the sessions in person.

### 2.3. Measures

The analysts completed two different sections.

#### 2.3.1. First section: *ad-hoc* constructed questionnaire

A special form was constructed with questions for analysts concerning the transition to remote therapy and the return to in-person therapy. The questionnaire covered the following areas: use of remote treatment; analysts’ acceptance; patients’ compliance; appropriateness of the therapeutic relationship and any difficulties encountered; effects on the therapeutic process; effects on the treatment also in relation to the disorder and type of patient; responses to return to in-person setting; subjective findings.

The questionnaire can be found in Appendix A.

#### 2.3.2. Second section: short *ad-hoc* survey

The form was constructed to collect information on the patient’s age, gender, type of problem, and duration of treatment. In addition, questions were formulated on a 5-point Likert scale concerning the patient’s family structure, work, and relational life.

#### 2.3.3. Interpretive and supportive technique scale

The ISTS measures the clinician’s therapeutic technique. Therapist technique refers to the technical procedures used to facilitate therapeutic change. The Interpretive and Supportive Techniques Scale, consisting of 14 items, quantifies the therapist’s degree of acceptance of the strategies provided in supportive and interpretive psychotherapies. It also indicated the amount of interpretive and supportive techniques provided. The 14 items – ranging from 0 (no emphasis) to 4 (great emphasis) – cover a range of interpretive and supportive common to different dynamic psychotherapies (Ogrodniczuk and Piper, 1999). In the present study, Cronbach’s alpha of the ISTS total score of the sample was considered good (α = 0.83).

#### 2.3.4. Working alliance inventory–short–therapist

The Working alliance inventory–short–therapist (WAI-S-T) (Horvath and Greenberg, 1989) validated Italian version was used (Lingiardi, 2002). evaluates the levels of the therapeutic alliance between patients and psychotherapists, from the psychotherapist’s standpoint. It consists of 12 items – measured on a 7-step Likert scale from 1 = never to 7 = always – assessing three key aspects of the therapeutic alliance: (a) agreement on the tasks of therapy, (b) agreement on the goals of therapy and (c) development of an affective bond. Moreover, the scale captures three dimensions: emotional bonding, and the level of agreement on therapy tasks and goals. In the current study, Cronbach’s alpha of the *WAI-S-T* total score of the sample was considered very good (α = 0.89).

#### 2.3.5. Relationship questionnaire

The Relationship questionnaire (RQ) (Bartholomew and Horowitz, 1991; Carli, 1995) provides a measure of the four attachment categories: secure, fearful, preoccupied and dismissing. It is a single-item measure, consisting of four short paragraphs, each of which describes a prototypical attachment pattern, applied to close relationships in adulthood. There are two parts, RQ1 and RQ2. In the first part, RQ1, participants are asked to select a paragraph-long description that best describes them, without providing a numerical rating. The essential statements for RQ1 are as follows. Secure attachment: “It is easy for me to become emotionally close to others. I feel comfortable depending on them and having them depend on me. I do not worry about being alone or that others will not accept me.” Fearful attachment: “I do not feel comfortable approaching others, I want emotionally close relationships, but I find it difficult to trust others completely or depend on them. I am afraid of being hurt if I allow myself to get too close to others.” Preoccupied attachment: “I do not feel comfortable getting close to others. I desire emotionally close relationships, but I find it difficult to trust others completely or to depend on them. I am afraid of being hurt if I allow myself to get too close to others.” Dismissing attachment: “I feel comfortable without close emotional relationships. It is very important for me to feel independent and self-sufficient, and I prefer not to depend on others or for others to depend on me.” In the second part, RQ2, participants are asked to rate their agreement with each prototype on a 7-point scale. The highest rating of the four attachment prototypes is used to classify the participants into an attachment category (Bartholomew and Horowitz, 1991). The RQ evidenced good construct, convergent, and divergent validity (Bartholomew and Horowitz, 1991). In the current study, Cronbach’s alpha of the RQ total score of the sample was considered good (α = 0.74).

Furthermore, for the present study, dichotomous classification was decided by dividing the subjects according to the secure and insecure attachment styles (fearful, preoccupied and dismissing).

#### 2.3.6. Personality matching anaclitic and introjective

The patients’ personality orientation was assessed using the Prototype Matching of Anaclitic-Introjective Personality Configuration (PMAI; Werbart and Levander, 2016). It is a clinician report form that presents prototypes of the anaclitic and introjective personality orientation. It consists of two items (one related to the predominantly anaclitic personality configuration or the introjective one) on a 5-step Likert scale (from 1 = poor/no match to 5 = very good match). The prototype matching method generates both categorical and dimensional ratings. Psychoanalysts were asked to rate how well their patients matched each prototype and to specify which of the two prototypes best matched the patient’s personality orientation. As we aimed to compare anaclitic and introjective participants, the results of the PMAI were used to classify participants into predominantly anaclitic or predominantly introjective orientation. Cases were classified as either anaclitic or introjective, based on the highest score on one of the two dimensions and based on categorical self-assessment in cases where both dimensions had the same score. In the current study, Cronbach’s alpha of the PMAI total score of the sample was considered good (α = 0.74).

## 3. Data analysis

Analyses were performed using SPSS 28.0 statistical software.

Skewness and kurtosis analyses were used to evaluate the normality of the distribution of the sample. All the variables, except for personality configuration, resulted within the acceptable range between −2 and + 2 (Podsakoff et al., 2003).

Descriptive statistics were used in the first section and the Chi-square test, paired sample *t*-test, ANOVA and Pearson r correlation were used in the second section.

Particularly, *T*-test and Chi-square test were used to evaluate the differences between patients with easy transition and patients with difficult transition to online psychotherapy on socio-demographics characteristics, type of issue, attachment style, levels of psychotherapeutic alliance, and psychotherapeutic technique (supportive and interpretive styles). *T*-test and Chi-square test were also performed to compare the effect of personality configuration (introjective vs. anaclitic) on the levels of the psychotherapeutic alliance, attachment style, and psychotherapeutic technique.

ANOVA was used to evaluate differences between patients with easy or difficult transitions and remote work or sessions in person in therapeutic alliance and technique.

Pearson r correlation was used to examine possible associations between attachment styles, levels of the therapeutic alliance, and psychotherapeutic technique.

A power analysis was conducted. This study in the second section was limited to 20 analysts; for *t*-test with 0.05 alpha level and 0.5 effect size, the statistical power was 33%. Accordingly, the results of this preliminary investigation must be interpreted with caution.

## 4. Results

### 4.1. First section

Descriptive statistics were derived from a total sample of 71 analysts.

During the acute phase of the pandemic, 100% of the analysts used remote therapy with at least one patient, including 47.8% with all or almost all patients. Various audio-visual tools were used: in 73.2% of the cases audio or video interviews, in 25.4% by telephone and 1.4% by written exchanges. Analysts rated the use of remote therapy as very helpful in 44.8%, fairly helpful in 44% and average or not very helpful in 10.2%. About 60% of the analysts reported that the patients had accepted the change and 83.1% had a good acceptance of remote therapy; only 24.1% of the patients did not accept the shift to remote therapy. In most cases (77%), analysts found that patients felt welcome, and that continuity was maintained.

30% were afraid that remote therapy would increase emotional distance and 22.4% that it would alter the analyst’s identity in his/her usual setting to a great or moderate extent; however, in 79.1% remote therapy was seen as a way of meeting patients’ needs.

28.3% of the analysts considered remote therapy to be a natural adaptation process without consequences, while 71.7% believed it led to some consequences. 19.4% of the analysts had a lot or enough ethical concerns (privacy, etc.).

85.9% of the analysts considered active listening necessary (with an average intensity ranging from very necessary), particularly with specific categories of patients: Attachment problems (56.7%), dependent traits (31.3%), and a tendency toward autonomy (11.9%). In 93.2%, The containment function was on average, fairly or very much activated.

All analysts reported little or no loss of human contact while 75.9% reported much or quite a lot of increase in splitting defences.

Concerning the subjective aspects of the analyst, 50% of them report that the experience of the pandemic for the patients was quite or very traumatic. Concerning the therapeutic function, for 79.6% the therapeutic continuity allowed a great deal of or fairly good containment of anxiety. Concerning therapeutic activity, 20.6% of the analysts were able to initiate new treatments, of which 24.1% to cope with pandemic-related issues and 50% mainly related to other problems.

About the therapeutic relationship, for 60.1% the return to in-person setting strengthened the relationship very or fairly much, however with some difficulty in re-establishing the sense of security (43.6%); 33.4% of the analysts reported strong or fairly marked emergence of repressed contents, improving therapeutic processing. None refused to return to psychotherapy in person.

### 4.2. Second section

#### 4.2.1. Preliminary analysis

For data analysis in the second section, two groups were created based on the evaluation given by the analysts: patients with easy transition (*N* = 20) and patients with difficult transition (*N* = 20). In patients with easy transition, 56.4% were female and 43.6% were male, while in patients with difficult transition 31.8% were female, 59.1% were male and 9.1% were attributed to the third gender. The differences between the two groups for gender were not significant (*Χ*^2^ = 3.15; *p* = 0.20). Patients with easy transition had a mean age of 40.29 years old (SD = 15.8) and patients with difficult transition had a mean age of 39.86 years old (SD = 10.99); there were no significant differences with respect to age (*t* = 0.11; *p* = 0.91).

In the group of patients with easy transition, 5.1% started the therapy recently, 94.9% had been in treatment for a long time; in the group of patients with difficult transition, 45.5% started the therapy recently, 54.5% had been in treatment for a long time. The difference between the two groups was statistically significant (*Χ*^2^ = 14.47; *p* < 0.001): patients with easy transition had a higher percentage who had already started therapy for a long time compared to patients with difficult transition.

In the group of patients with easy transition, 41% undertook therapy for problems evaluated as neurotic, 30% for personality disorders, 12.8% for psychotic and 15.4% for family problems; in the group of patients with difficult transition, 36.4% undertook therapy for problems evaluated as neurotic, 50% personality disorders, 4.5% psychotic and 9.1% about family. The differences between the two groups were not significant (Fisher’s exact test = 2.86; *p* = 0.41).

Finally, patients with easy transition had a higher score in the structured family life category than patients with difficult transition (*t* = 2.57; *p* = 0.013). On the other hand, there were no significant differences with respect to the scores relating to structured working life (*t* = 0.65; *p* = 0.51) and structured relational life (*t* = 1.72; *p* = 0.08) categories.

#### 4.2.2. Differences in attachment style, personality configuration, level of psychotherapeutic alliance, and psychotherapeutic technique

*T*-test and Chi-square (or Fisher exact test) were used to evaluate the differences between the two groups in attachment style, personality configuration, level of psychotherapeutic alliance, and psychotherapeutic technique.

The results showed a significant association between the type of transition to online psychotherapy and attachment style [*Χ*^2^ (1;37) = 5.49; *p* = 0.033]. Patients with difficult transition to online psychotherapy had a more insecure attachment style: 95% of patients with difficult transition had an insecure attachment and 5% had secure attachment while 65.7% of patients with easy transition had an insecure attachment and 35.3% had a secure attachment.

No significant associations emerged between the type of transition to online psychotherapy and personality configurations [*Χ*^2^ (1;40) = 0.1; *p* = 1.00]. In both groups, half of the patients were assessed as having an introjective personality configuration and half as having an anaclitic personality configuration.

*T*-test showed a significant difference between patients with easy transition and patients with difficult transition to online psychotherapy (see Table 1). Patients with difficult transition had a higher score on the RQ Dismissing scale than patients with easy transitions. Moreover, at the level of a tendency toward significance, patients with difficult transition had a higher score on the RQ Fearful scale than patients with easy transition.

**Table 1:** Differences between patients with easy transition and patients with difficult transition on attachment scales, levels of therapeutic alliance, and psychotherapeutic technique.

	Patients with easy transition (*N* = 20)	Patients with difficult transition (*N* = 20)	*t*	*p*	*d*
WAI-T	4.44 (0.74)	4.38 (0.74)	0.25	0.80	
ISTS	21.45 (9.04)	21.60 (8.81)	−0.05	0.95	
RQ Secure	2.41 (1.00)	2.60 (1.56)	−0.42	0.67	
RQ Dismissing	2.47 (1.17)	3.45 (1.46)	−2.21	0.034*	
RQ Preoccupied	3.41 (0.93)	3.80 (1.57)	−0.88	0.38	
RQ Fearful	2.94 (1.24)	3.90 (1.61)	−1.98	0.055+	

*p < 0.05, **p < 0.01, ***p < 0.001.

No significant differences were revealed between patients with easy transition and difficult transition in the level of psychotherapeutic alliance and psychotherapeutic technique.

Subgroups were also created based on the setting – remote work and return to in-person. The differences for the four subgroups based on the analysts’ evaluations (20 patients with easy transition to remote work, 20 patients with easy transition back to in-person setting, 19 patients with difficult transition to remote work and 20 patients with difficult transition back to in-person setting) regarding therapeutic alliance or in the use of interpretive and supportive techniques were evaluated through univariate ANOVA. The results indicated a significant main group effect for the level of therapeutic alliance [*F*(3,79) = 8.16; *p* < 0.001]. Bonferroni post-doc test indicated that patients with easy transition to remote work had higher scores of therapeutic alliance both than patients with difficult transition to remote work (*p* = 0.002) and patients with difficult transition back to in-person setting (*p* = 0.017); patients with easy transition back to in-person setting had higher scores of therapeutic alliance both than patients with difficult transition to remote work (*p* = 0.001) and patients with difficult transition back to in-person setting (*p* = 0.014). No significant differences emerged between patients with easy transition back to in-person setting and patients with easy transition to remote work (*p* = 1.00) and between patients with difficult transition back to in-person setting and patients with difficult transition to remote work (*p* = 1.00).

Regarding the use of interpretive and supportive techniques, we found no significant main group effect [*F*(3,77) = 0.03; *p* = 0.99].

The differences for the personality configuration were evaluated, considering the two groups of patients with an introjective personality configuration (*N* = 21) and patients with an anaclitic personality configuration (*N* = 19).

Furthermore, *t*-test and chi-square were used to evaluate possible associations between personality configurations and levels of therapeutic alliance, psychotherapeutic technique, and attachment style. Fisher exact test showed no significant differences between personality configurations and attachment styles [(1;37) = 0.43; *p* = 0.68]. Both for patients with an introjective configuration (85%) and for patients with an anaclitic configuration (76.5%) the insecure attachment style was prevalent.

*T*-test showed no significant differences between introjective and anaclitic personality configurations in RQ scales, levels of therapeutic alliance, and psychotherapeutic technique (see Table 2).

**Table 2:** Differences between introjective and anaclitic personality configurations on attachment scales, levels of therapeutic alliance and psychotherapeutic technique.

	Patients with anaclitic personality (*N* = 20)	Patients with introjective personality (*N* = 20)	*t*	*p*	*d*
WAI-T	4.30 (0.79)	4.50 (0.68)	−0.84	0.40	
ISTS	22.18 (7.70)	20.92 (9.92)	0.44	0.66	
RQ Secure	2.35 (1.22)	2.65 (1.42)	−0.67	0.50	
RQ Dismissing	3.47 (1.50)	2.60 (1.23)	1.93	0.061	
RQ Preoccupied	3.41 (1.54)	3.80 (1.10)	−0.89	0.38	
RQ Fearful	3.47 (1.87)	3.45 (1.19)	0.03	0.96	

*p < 0.05, **p < 0.01, ***p < 0.001.

Finally, through Pearson correlation analyses, we evaluated possible associations between RQ attachment scales, levels of therapeutic alliance, and psychotherapeutic technique. The results showed significant associations: the level of the therapeutic alliance was positively correlated to the RQ Secure scale (*p* = 0.005) and negatively correlated to RQ Dismissing scale (*p* = 0.022) (see Table 3).

**Table 3:** Correlation between attachment scales, levels of therapeutic alliance, and psychotherapeutic technique.

	(1)	(2)	(3)	(4)	(5)	(6)
WAI-SR-T (1)	–	0.16	0.45**	−0.37*	−0.16	0.00
ISTS (2)		–	0.16	−0.16	0.05	0.11
RQ Secure (3)			–	−0.31	−0.31	−31
RQ Dismissing (4)				–	0.17	0.19
RQ Preoccupied (5)					–	0.60***
RQ Fearful (6)						–

*p < 0.05, **p < 0.01, ***p < 0.001.

## 5. Discussion

Regarding the part of the general questionnaire about how analysts perceived the transition from in-person to remote treatment for themselves and their patients, an analysis of the results reveals an overall positive picture. According to the responders, most patients accepted this transition, experienced it positively in most cases and felt they could maintain the continuity of the therapeutic work. For their part, most analysts feel that they succeeded in meeting their patients’ needs, making them feel welcome and contained. However, concerns experienced by analysts regarding the setting modification and their own analytical identity also emerged, as well as the fear of creating greater emotional distance with the patient.

Regarding the remote analytical process, most analysts believe that online treatment had relevant technique-related consequences, while a third of them considered online therapeutic work to be a smooth adaptation to the lockdown conditions imposed by the pandemic, agreeing with Bolognini’s (2020) observations. According to most responders, it was necessary to enhance the listening and holding functions in the therapeutic work, mainly with some categories of patients with attachment and dependency-related problems. It is also interesting to note that many of the analysts pointed out an increase in splitting defenses recruited by patients. Despite these variations, all the analysts agreed that there was no loss of human contact with patients and that the continuity of the therapeutic work provided by the online treatment enabled the containment of anxiety (as also noted by Altman, 2020; Gampel, 2020) as even with regard to the traumatic experience of the pandemic experienced by patients.

Regarding the return to in-person setting, this step also does not seem to have entailed difficulties according to the analysts. In fact, it strengthened the therapeutic relationship in the patients according to most responders, although with some difficulties in re-establishing a sense of safety compared to sessions in person. Finally, it is interesting to note that according to about one third of the responders, the return to in-person setting allowed the emergence of unconscious contents that had not emerged in remote treatment (as also observed in the recent literature). This finding is consistent with the analysts’ observation of the emergence of splitting processes in online treatment.

The results discussed above are in line with other studies. In particular Aafjes-van Doorn et al. (2021b) in a two-stage study – during the first weeks of lockdown and after about 2 months – showed that the majority of analysts considered online therapy in the follow-up as similar to in-person treatment, feeling positively connected and authentic in their work with their patients and overcoming the initial concerns about not feeling competent and experienced in the initial stage. Békés et al.’s (2020) study that compared analysts’ perceptions in in-person and online treatments also shows that most of them felt they were equally connected and authentic with their patients in both therapies. In this respect, Humer et al.’ (2020) study showed how a large number of the psychotherapists interviewed considered the transition to online therapy via videoconferencing to be better than expected. Finally, other studies showed that online therapy has made it possible to establish a relationship with the patient that maintains therapeutic continuity (Ehrlich, 2021; Nicolò, 2021).

On the other hand, the part of the questionnaire, which compared patients with easy transition to online treatment with those with difficult transition and aimed at investigating whether and how the psychoanalysts had experienced this transition in the patients, produced interesting results. First of all, we can consider no significant variables such as gender and age. Particularly, we had thought that age could introduce important differences regarding disposition to transition. We do know that young people – also among the psychotherapists – are generally more accustomed to using devices which connect people, and we thought that this situation could be experienced as a challenge (Aafjes-van Doorn et al., 2022). Diagnosis also did not emerge as a variable involved in determining significant differences between the two groups of patients. As we somewhat expected, the length of the treatment that had already been done facilitated an easy transition to the online approach. Indeed, the length of the treatment is often correlated to a stable alliance (Heinonen et al., 2022).

The use of some tools allowed us to better articulate these first comments about the results. In particular, while the analysis of personality configurations (anaclitic, introjective) did not show significant differences between the groups, contrary to expectations (Werbart et al., 2022b), the analysis of attachment styles showed significant differences overall, highlighting a higher frequency of insecure attachment styles in patients with difficult transition to remote therapy than in those with easy transition. In this respect, insecure attachment style emerged as a risk factor for coping with the transition to remote therapy, confirming the vulnerability aspects inherent in insecure vs. secure attachment styles (Mikulincer and Shaver, 2012; Riva Crugnola et al., 2021; Aafjes-van Doorn et al., 2021a). Moreover, among attachment styles, the significant differences that emerged between the two groups considered concerned the dismissing attachment style and, with a tendency toward significance, the fearful one that involves both anxiety and avoidance. Regarding the dismissing style, we can hypothesize that this style, which expresses difficulties with respect to the intimacy of relationships, made it difficult to adapt to the new relational mode proposed by the analysts. Regarding patients with fearful style expressing both anxiety and avoidance, the online approach could lead to the perception of “the inanimate third” – the electronic device – in the therapeutic process (Ferber et al., 2022). The presence of the “analytic third” was assumed by Ogden (2004). He refers to the connection created through the unconscious life of the analytic pair. On the contrary, “the inanimate third” emphasizes how the objectivity of the electronic device is in opposition to the subjective emotional processes involved in the psychoanalytic process.

As to the therapeutic alliance, its particular importance is confirmed. Indeed, our results show that it favored both the transition to online treatment and the return to in-person sessions. Furthermore, the therapeutic alliance is positively correlated to a secure, non-problematic attachment style comparing to those mentioned above (dismissing and anxious). In other words, the therapeutic alliance is confirmed as a construct at the basis of the psychotherapeutic process (Safran and Muran, 2000; Oasi, 2015).

Finally, the lack of distinction between types of psychotherapeutic intervention – supportive vs. expressive – could be in line with the hypothesis that remote treatments tend to “flatten the differences” (Probst et al., 2020), but it could also indicate that the working-through and supportive interventions are parts of a single process that is modeled on different levels of subjective needs in the patient and in the alternation we studied. This can be thought of as characterizing the adaptation process, which involves a partial temporary regression that occurs at critical moments (Roussillon, 2020 Guignard and Diatkine, 2021) and then triggers growth processes.

Besides theoretical considerations, generally speaking, this study highlights that the quality of the psychoanalytic process is involved in different ways during the transition from the consulting room to the online setting, but further research is needed for understanding how individual differences can intervene significantly (Johnson et al., 2022). Although some results are promising, currently even important and validated constructs such as attachment or personality orientations (see this study and Werbart et al., 2022b) do not give enough certainty. It might be important to take into consideration: the type of patients – children or adolescents vs. adult or older people (Erlandsson et al., 2022) and/or psychotherapist orientation – for ex., psychodynamic vs. cognitive (Sachs et al., 2022).

### 5.1. Limits and future directions

The study is not without limitations. The interviews were only addressed to the analysts and not directly to the patients. Analysts had low familiarity with the evaluation tools of the second section. Moreover, the data were collected based on the perceptions of the analysts involved. Since there were no collected data from patients, it would be important in future studies to reproduce the study from patients’ perspectives. The number of the responders was low and does not allow for a generalization of data. The results obtained in this study are to be considered exploratory and preliminary. Replication of the study with a larger sample is deemed necessary and unavoidable. A final limitation concerns the validation of instruments. Of the 4 included instruments, only RQ (Carli, 1995) and WAI-S -T (Lingiardi, 2002) have Italian validation. Regarding future directions, despite the overall positive assessment outlined by the analysts in our study about the use of remote treatment during the pandemic, many analysts also highlighted concerns about this use especially with regards to distance which can intensify defenses. At the same time, the study showed some characteristics of the patients that made the transition to remote treatment more difficult and requiring more attention in clinical practice. In any case, we faced a new frame of working with the patients which in part did not disappear once back on the coach. Further studies about how the training in some scientific community is held online (Moshtagh, 2020) and whether a specific training to conduct teleanalysis is required (Ahlström et al., 2022) are recommended.

## Data availability statement

The raw data supporting the conclusions of this article will be made available by the authors, without undue reservation.

## Ethics statement

Ethical approval was not provided for this study on human participants because the research was authorized by the President of the Italian Psychoanalytic Society within which it was performed and followed the principles of the Declaration of Helsinki. The patients/participants provided their written informed consent to participate in this study.

## Author contributions

LLR, AW, OO, and CRC planned, designed the present study, wrote, and reviewed the manuscript. FDS and EI wrote, reviewed the manuscript, and analyzed the data. MG participated in data collection. All authors have given final approval of the version to be published.

## Funding

The work was supported by the Grant assigned by the International Psychoanalytical Association (IPA), December 15, 2021, to the project: Transition to remote therapy and back, personality orientation and attachment style. 2021 Research Grant Application Number: 6. Moreover, the work was supported by the Fund for Psychoanalytic Research of the American Psychoanalytic Association dated May 26, 2022, and the International Psychoanalytical Association Research Grant dated October 12, 2022.

## Conflict of interest

The authors declare that the research was conducted in the absence of any commercial or financial relationships that could be construed as a potential conflict of interest.

## Publisher’s note

All claims expressed in this article are solely those of the authors and do not necessarily represent those of their affiliated organizations, or those of the publisher, the editors and the reviewers. Any product that may be evaluated in this article, or claim that may be made by its manufacturer, is not guaranteed or endorsed by the publisher.
