# Supplementary material for: Understanding psychoanalytic work online and back to the couch in the wake of the COVID-19 pandemic: an investigation among Italian psychoanalysts
Source: Front Psychol. 2023 Jun 22;14:1167582. doi: 10.3389/fpsyg.2023.1167582 (PMC10324410; doi:10.3389/fpsyg.2023.1167582)
Supplement: Supplementary file 1 [file Data_Sheet_1.pdf]

Supplementary Material

Understanding psychoanalytic work online and back to the couch in the wake of the COVID-19 pandemic: An investigation among Italian Psychoanalysts

Licia Lea Reatto[1*], Andrzej Werbart[2], Osmano Oasi[3], Francesca De Salve[3], Elena Ierardi[4*], Mattia Giordano[3], Cristina Riva Crugnola[4]

[1]IPA and Italian Psychoanalytic Society, Italy

[2]Department of Psychology, Stockholm University, Stockholm, Sweden

[3]Department of Psychology, Catholic University of the Sacred Heart, Milan, Italy

[4]Department of Psychology, University of Milano-Bicocca, Milan, Italy

*** Correspondence:**
Licia Reatto: liciareatto@hotmail.com

Elena Ierardi: elena.ierardi@unimib.it

First section: ad-hoc constructed survey

1. During the acute period of Covid-19 did you use remote therapy?
2. During the acute period of Covid-19 did you use remote therapy with most of the patients?
3. During the acute period of Covid-19 did you use remote therapy only for the special needs of patients (e.g., travel, illness, etc.)?
4. During the acute period of Covid-19 did you use remote therapy in conditions related to the pandemic?
5. During the acute period of Covid-19 did you use remote therapy?
6. In conducting therapy remotely, which modalities did you prefer?
7. When switching to remote therapy, please indicate how often, in your relationship with the patient, you have observed the following reactions: (answer on the scale indicated from 1 to 5, where 1 = very little/not at all; 2 = very little; 3 = moderately; 4 = quite a lot; 5 = very much/always):
    - Patients welcomed the opportunity to continue
    - They had no difficulty accepting the change
    - Patients felt welcomed
    - The continuation of therapy under changed conditions helped them to cope
    - The therapeutic relationship could maintain its continuity
    - The elaborative process could take its course

- The change sometimes created a sense of distance
- Physical distance hindered patients' reliance
- The poignancy of the real situation interfered with the associative process
- Patients felt stimulated to boost their resources in the face of the problematic situation
- More listening had to be activated
- In general, the common dangerous situation facilitated listening
- The space for processing could be preserved
- Concern for each other's safety prevailed at times
- This fear interfered with the processing capacity
- The dangerous situation sometimes made the meeting difficult
- Willingness to associate tended to decrease
- You had the impression that the change of setting favored the therapeutic process
- That the patients felt more welcome
- The absence of body contact interfered with the therapeutic encounter

8. Was it necessary to activate more listening with certain categories of patients?
    - Patients with marked dependency traits
    - Patients with a prevalent tendency toward autonomy
    - Patients with attachment problems
9. Was distance therapy a novelty?
10. Did you feel the use of this medium was useful?
11. You were afraid it would increase the emotional distance?
12. You feared that your identity as an analyst in its usual setting might be altered?
13. Did you think of it as a way to meet the needs of patients?
14. You felt it was a natural process of adaptation without consequences
15. You had ethical concerns (privacy etc.)?
16. Now we ask you to reflect on what happened during the remote therapy and quantify the following statements on a scale of 1 to 5 (1 = very little-not/ at all; 2 = very little; 3 = average; 4 = quite a lot; 5 = very-almost/ always):
    - The emergency made the containment function prevail in the relationship
    - It favored elaboration by bringing out removed areas
    - The alteration of reality conditions favored the reactivation of traumatic cores, and removed memories, with influence on the therapeutic relationship
    - The course of the therapeutic relationship was sometimes made difficult by the frequent variation of the elements of reality
    - The anxiety of loss of the relationship increased in the patients
    - Processing became more problematic
    - Variation sometimes had a positive influence on processing, e.g., by an increasing alliance
17. Did the pandemic situation leave significant traces in the dream material?
18. During the pandemic, we were personally involved:
    - You sometimes felt it difficult to preserve your internal setting regarding the development of the therapeutic relationship
    - In the altered situation, you sometimes found it difficult to follow the development of the transference

- You sometimes found it difficult to adequately recognize counter-transference movements
- You found it sometimes difficult to follow the associative process (fluctuating attention)

19. Thinking back to the characteristics of the patients during the pandemic period, to what extent did you witness:
    - Increasing personal resources
    - Losing of human contact
    - Strengthening of phobic cores
    - Increasing splitting aspects
20. Did the transition, through physical distance, sometimes allow for greater visual proximity (presence of each other's family environments, greater mutual knowledge of aspects of real life)?
21. In your experience this:
    - Did it foster a climate of greater closeness
    - Did it interfere with the therapeutic situation
    - Did the patients feel reassured by your availability
22. For patients who presented with psychotic anxiety, how much did the presence of external difficulties seem to attenuate projective tendencies?
23. Were you able to undertake new therapies during the transition?
24. Indicate to what extent (1 = very little/not at all; 2 = very little; 3 = moderately; 4 = quite a lot; 5 = very much/always):
    - For problems related to the pandemic situation (e.g., distance, fear of contact, etc., etc.)
    - For independent problems
25. Have interruptions or suspensions due to Covid-19 occurred frequently in therapy?
26. With the pandemic subsiding, and the protection afforded by vaccinations, have you been able to resume activity back in person?
27. Were there any patients who did not accept the remote transition?
28. Have some patients not accepted return attendance?
29. In the phase of return to work in person, partial or total, how much:
    - Patients appeared to react with relief to the resumption of work in person
    - The therapeutic relationship struggled to regain its stability
    - Found no change compared to remote work
    - The sense of a common experience strengthened the relationship
    - For some patients, it was particularly difficult to regain trust
    - Patients with a predominantly autonomous tendency were better off working at a distance
    - For patients with psychotic functioning did the return to the practice increase relationship anxieties
    - Did you experience sudden improvements in fronting the emergency?
30. In your experience, do you think that the pandemic may have been a traumatic event for the patient?
31. And what about you?
32. Do you think that therapeutic continuity and the participation of the social context may have had a restraining effect on the anxiety mobilized by the pandemic?
33. Was the therapeutic relationship strengthened in the recovery phase?

34. Was it difficult to restore a sense of security?
35. Were new, previously unexpressed contents able to emerge, which fostered the therapeutic relationship?
36. Do you remember any dreams during the period of back in presence?
